# Crystal structure of *N*-(1-allyl-3-chloro-1*H*-indazol-5-yl)-4-methyl­benzene­sulfonamide

**DOI:** 10.1107/S1600536814018194

**Published:** 2014-08-23

**Authors:** Hakima Chicha, El Mostapha Rakib, Mohamed Chigr, Mohamed Saadi, Lahcen El Ammari

**Affiliations:** a Laboratoire de Chimie Organique et Analytique, Université Sultan Moulay Slimane, Faculté des Sciences et Techniques, Béni-Mellal, BP 523, Morocco; b Laboratoire de Chimie du Solide Appliquée, Faculté des Sciences, Université Mohammed V-Agdal, Avenue Ibn Battouta, BP 1014, Rabat, Morocco

**Keywords:** crystal structure, benzene­sulfonamides, biological activity, hydrogen bonding

## Abstract

The 3-chloro-1*H*-indazole system in the title mol­ecule, C_17_H_16_ClN_3_O_2_S, is almost planar, with the largest deviation from the mean plane being 0.029 (2) Å for one of the N atoms. This system is nearly perpendicular to the allyl chain, as indicated by the C—C—N—N torsion angle of −90.1 (6)° between them. The allyl group is split into two fragments, the major component has a site occupancy of 0.579 (7). The indazole system makes a dihedral angle of 47.53 (10)° with the plane through the benzene ring. In the crystal, mol­ecules are connected by N—H⋯O and C—H⋯O hydrogen bonds, forming a three-dimensional network.

## Related literature   

For the biological activity of sulfonamides, see: El-Sayed, *et al.* (2011 ▶); Mustafa *et al.* (2012 ▶); Scozzafava *et al.* (2003 ▶). For similar compounds, see: Abbassi *et al.* (2012 ▶, 2013 ▶); Chicha *et al.* (2014 ▶).
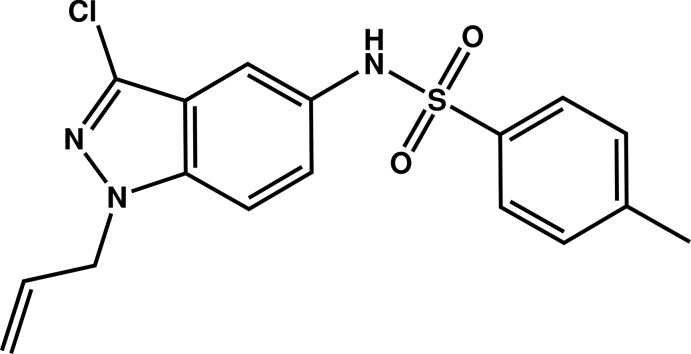


## Experimental   

### Crystal data  

C_17_H_16_ClN_3_O_2_S
*M* *_r_* = 361.84
Orthorhombic, 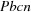

*a* = 8.1736 (12) Å
*b* = 22.504 (4) Å
*c* = 19.279 (3) Å
*V* = 3546.2 (10) Å^3^
*Z* = 8
Mo *K*α radiation
μ = 0.35 mm^−1^
*T* = 296 K
0.40 × 0.36 × 0.31 mm

### Data collection  

Bruker X8 APEX diffractometer
Absorption correction: multi-scan (*SADABS*; Sheldrick, 2008 ▶) *T*_min_ = 0.693, *T*_max_ = 0.747
18362 measured reflections
3621 independent reflections
2327 reflections with *I* > 2σ(*I*)
*R*_int_ = 0.051

### Refinement  

*R*[*F* ^2^ > 2σ(*F* ^2^)] = 0.043
*wR*(*F* ^2^) = 0.123
*S* = 1.02
3621 reflections
225 parameters
4 restraints
H-atom parameters constrained
Δρ_max_ = 0.26 e Å^−3^
Δρ_min_ = −0.26 e Å^−3^

### 

Data collection: *APEX2* (Bruker, 2009 ▶); cell refinement: *SAINT* (Bruker, 2009 ▶); data reduction: *SAINT*; program(s) used to solve structure: *SHELXS97* (Sheldrick, 2008 ▶); program(s) used to refine structure: *SHELXL97* (Sheldrick, 2008 ▶); molecular graphics: *ORTEP-3 for Windows* (Farrugia, 2012 ▶); software used to prepare material for publication: *PLATON* (Spek, 2009 ▶) and *publCIF* (Westrip, 2010 ▶).

## Supplementary Material

Crystal structure: contains datablock(s) I. DOI: 10.1107/S1600536814018194/tk5336sup1.cif

Structure factors: contains datablock(s) I. DOI: 10.1107/S1600536814018194/tk5336Isup2.hkl

Supporting information file. DOI: 10.1107/S1600536814018194/tk5336Isup3.cml

**Fig. 1.**
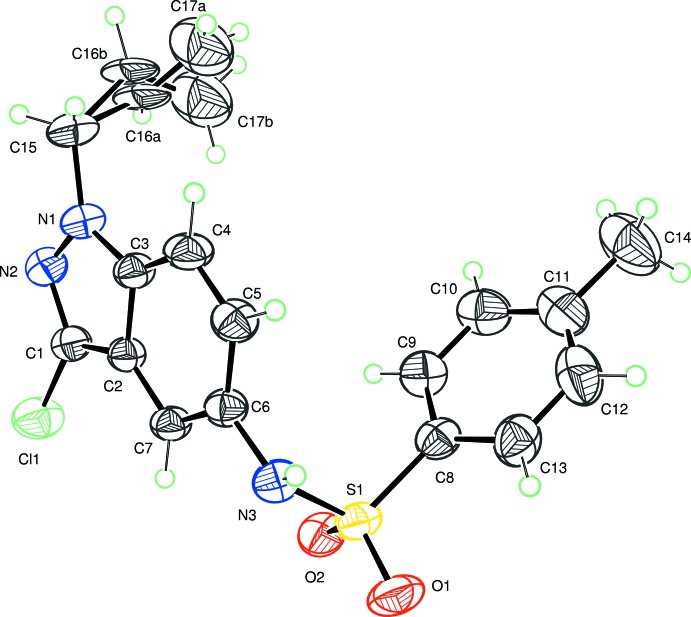
Molecular structure of the title compound with the atom-labelling scheme. Displacement ellipsoids are drawn at the 50% probability level. H atoms are represented as small circles.

. DOI: 10.1107/S1600536814018194/tk5336fig1.tif
Mol­ecular structure of the title compound with the atom-labelling scheme. Displacement ellipsoids are drawn at the 50% probability level. H atoms are represented as small circles.

**Fig. 2.**
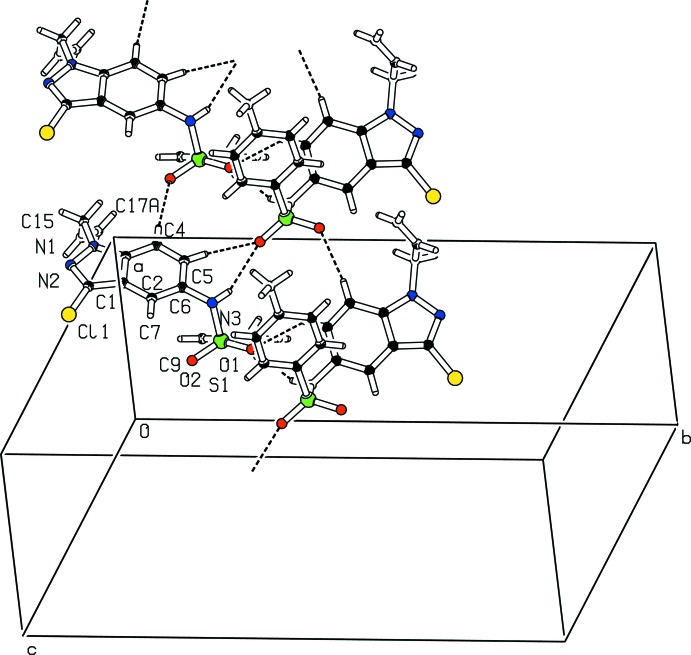
Crystal structure of the title compound, showing molecules linked by N3–H3N···O1, C5–H5···O1 and C4–H4···O2 hydrogen bonds between molecules.

N . DOI: 10.1107/S1600536814018194/tk5336fig2.tif
Crystal structure of the title compound, showing mol­ecules linked by N3–H3*N*⋯O1, C5–H5⋯O1 and C4–H4⋯O2 hydrogen bonds between mol­ecules.

CCDC reference: 1018456

Additional supporting information:  crystallographic information; 3D view; checkCIF report

## Figures and Tables

**Table 1:** Hydrogen-bond geometry (Å, °)

*D*—H⋯*A*	*D*—H	H⋯*A*	*D*⋯*A*	*D*—H⋯*A*
N3—H3*N*⋯O1^i^	0.81	2.39	3.140 (3)	155
C4—H4⋯O2^ii^	0.93	2.44	3.364 (3)	171
C5—H5⋯O1^i^	0.93	2.58	3.282 (3)	132

Symmetry codes: (i) 
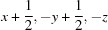
; (ii) 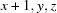.

## supplementary crystallographic information

### S2. Structural commentary

Sulfonamides are an important class of compounds which are widely used in the design of diverse classes of drug candidates (El-Sayed *et al.*, 2011; Mustafa *et al.*, 2012; Scozzafava *et al.*, 2003). Previously, we identified a series of indazoles bearing a sulfonamide moiety with good anti­proliferative activities (Abbassi *et al.*, 2012; Abbassi, *et al.* 2013; Chicha *et al.*, 2014).

The molecule of the title compound is built up from two fused five- and six-membered rings (N1 N2 C2 to C8) almost coplanar, with a maximum deviation of 0.029 (2) Å for N1 atom (Fig. 1). The dihedral angle between the indazol system and the plane through the benzene ring (C9 to C14) is of 47.53 (10)°. The allyl chain is perpendicular to the fused rings system as indicated by the C(16A)—C(15)—N(1)—N(2) torsion angle of -90.1 (6)°.

The cohesion of the crystal structure is ensured by N3–H3N···O1, C5–H5···O1 and C4–H4···O2 hydrogen bonds between molecules to form a three-dimensional network as shown in Fig. 2 and Table 1.

### S5. Synthesis and crystallization

A mixture of 1-allyl-3-chloro-5-nitro­indazole (1.22 mmol) and anhydrous SnCl_2_ (1.1 g, 6.1 mmol) in 25 ml of absolute ethanol was heated at 333 K for 6 h. After reduction, the starting material disappeared, and the solution was allowed to cool down. The pH was made slightly basic (pH 7–8) by addition of 5% aqueous potassium bicarbonate before extraction with ethyl acetate. The organic phase was washed with brine and dried over magnesium sulfate. The solvent was removed to afford the amine, which was immediately dissolved in pyridine (5 ml) and then reacted with 4-methyl­benzene­sulfonyl chloride (1.25 mmol) at room temperature for 24 h. After the reaction mixture was concentrated *in vacuo*, the resulting residue was purified by flash chromatography (eluted with ethyl acetate:hexane 2:8). The title compound was recrystallized from its ethanol solution. Yield: 65%, M.pt: 394 K.

### S6. Refinement

The reflections (002), (110), (021) and (020), probably affected by the beam stop, were removed from the final refinement. The refinement of the model, i.e. disordered allyl group, required constraints on the distance C15—C16—C17 and atomic displacements of allyl group. The H atoms were located in a difference map and treated as riding with C—H = 0.96 Å, C—H = 0.97 Å, C—H = 0.93 Å, and N—H = 0.81 Å for methyl, methyl­ene, aromatic CH and NH, respectively, and with *U*_iso_(H) = 1.2 *U*_eq_ (methyl­ene, aromatic, NH) and *U*_iso_(H) = 1.5 *U*_eq_ for methyl.

### Crystal data

**Table:**

C_17_H_16_ClN_3_O_2_S	*D*_x_ = 1.355 Mg m^−^^3^
*M**_r_* = 361.84	Melting point: 394 K
Orthorhombic, *P**b**c**n*	Mo *K*α radiation, λ = 0.71073 Å
Hall symbol: -P 2n 2ab	Cell parameters from 3621 reflections
*a* = 8.1736 (12) Å	θ = 2.8–26.4°
*b* = 22.504 (4) Å	µ = 0.35 mm^−^^1^
*c* = 19.279 (3) Å	*T* = 296 K
*V* = 3546.2 (10) Å^3^	Block, colourless
*Z* = 8	0.40 × 0.36 × 0.31 mm
*F*(000) = 1504	

### Data collection

**Table:**

Bruker X8 APEX diffractometer	3621 independent reflections
Radiation source: fine-focus sealed tube	2327 reflections with *I* > 2σ(*I*)
Graphite monochromator	*R*_int_ = 0.051
φ and ω scans	θ_max_ = 26.4°, θ_min_ = 2.8°
Absorption correction: multi-scan (*SADABS*; Sheldrick, 2008)	*h* = −9→10
*T*_min_ = 0.693, *T*_max_ = 0.747	*k* = −28→26
18362 measured reflections	*l* = −22→24

### Refinement

**Table:**

Refinement on *F*^2^	Secondary atom site location: difference Fourier map
Least-squares matrix: full	Hydrogen site location: inferred from neighbouring sites
*R*[*F*^2^ > 2σ(*F*^2^)] = 0.043	H-atom parameters constrained
*wR*(*F*^2^) = 0.123	*w* = 1/[σ^2^(*F*_o_^2^) + (0.0557*P*)^2^ + 0.7269*P*] where *P* = (*F*_o_^2^ + 2*F*_c_^2^)/3
*S* = 1.02	(Δ/σ)_max_ < 0.001
3621 reflections	Δρ_max_ = 0.26 e Å^−^^3^
225 parameters	Δρ_min_ = −0.26 e Å^−^^3^
4 restraints	Extinction correction: *SHELXL97* (Sheldrick, 2008), Fc^*^=kFc[1+0.001xFc^2^λ^3^/sin(2θ)]^-1/4^
Primary atom site location: structure-invariant direct methods	Extinction coefficient: 0.0026 (4)

### Special details

**Table:**

Geometry. All e.s.d.'s (except the e.s.d. in the dihedral angle between two l.s. planes) are estimated using the full covariance matrix. The cell e.s.d.'s are taken into account individually in the estimation of e.s.d.'s in distances, angles and torsion angles; correlations between e.s.d.'s in cell parameters are only used when they are defined by crystal symmetry. An approximate (isotropic) treatment of cell e.s.d.'s is used for estimating e.s.d.'s involving l.s. planes.
Refinement. Refinement of *F*^2^ against all reflections. The weighted *R*-factor *wR* and goodness of fit *S* are based on *F*^2^, conventional *R*-factors *R* are based on *F*, with *F* set to zero for negative *F*^2^. The threshold expression of *F*^2^ > σ(*F*^2^) is used only for calculating *R*-factors(gt) *etc*. and is not relevant to the choice of reflections for refinement. *R*-factors based on *F*^2^ are statistically about twice as large as those based on *F*, and *R*- factors based on all data will be even larger.

### Fractional atomic coordinates and isotropic or equivalent isotropic displacement parameters (Å^2^)

**Table:**

	*x*	*y*	*z*	*U*_iso_*/*U*_eq_	Occ. (<1)
C1	0.8260 (3)	−0.03622 (11)	0.08181 (12)	0.0516 (6)	
C2	0.8328 (3)	0.02554 (10)	0.06992 (11)	0.0427 (5)	
C3	0.9964 (3)	0.04008 (11)	0.08496 (12)	0.0481 (6)	
C4	1.0542 (3)	0.09849 (12)	0.07951 (13)	0.0570 (7)	
H4	1.1624	0.1081	0.0892	0.068*	
C5	0.9446 (3)	0.14053 (11)	0.05939 (13)	0.0532 (6)	
H5	0.9793	0.1798	0.0558	0.064*	
C6	0.7806 (3)	0.12692 (10)	0.04380 (11)	0.0444 (5)	
C7	0.7233 (3)	0.06945 (10)	0.04817 (11)	0.0445 (6)	
H7	0.6156	0.0602	0.0370	0.053*	
C8	0.6222 (3)	0.22836 (11)	0.14802 (13)	0.0503 (6)	
C9	0.6865 (3)	0.19241 (12)	0.19974 (14)	0.0628 (7)	
H9	0.6795	0.1513	0.1961	0.075*	
C10	0.7609 (4)	0.21805 (15)	0.25663 (15)	0.0741 (8)	
H10	0.8063	0.1939	0.2907	0.089*	
C11	0.7691 (4)	0.27915 (16)	0.26378 (16)	0.0756 (9)	
C12	0.7046 (4)	0.31338 (14)	0.21193 (19)	0.0830 (10)	
H12	0.7097	0.3545	0.2162	0.100*	
C13	0.6321 (3)	0.28965 (12)	0.15350 (16)	0.0669 (8)	
H13	0.5911	0.3141	0.1187	0.080*	
C14	0.8448 (5)	0.3061 (2)	0.32849 (19)	0.1179 (15)	
H14A	0.8818	0.2750	0.3586	0.177*	
H14B	0.7645	0.3298	0.3521	0.177*	
H14C	0.9358	0.3307	0.3156	0.177*	
C15	1.2392 (3)	−0.01960 (14)	0.12649 (15)	0.0755 (9)	
H15A	1.3111	0.0081	0.1029	0.091*	
H15B	1.2767	−0.0597	0.1172	0.091*	
C16A	1.2386 (8)	−0.0080 (6)	0.2012 (2)	0.0902 (19)	0.579 (7)
H16A	1.1739	−0.0334	0.2276	0.108*	0.579 (7)
C17A	1.3154 (12)	0.0324 (4)	0.2354 (5)	0.120 (2)	0.579 (7)
H17A	1.3823	0.0593	0.2122	0.144*	0.579 (7)
H17B	1.3039	0.0348	0.2833	0.144*	0.579 (7)
C16B	1.2961 (13)	−0.0101 (9)	0.1975 (3)	0.0902 (19)	0.421 (7)
H16B	1.3979	−0.0238	0.2119	0.108*	0.421 (7)
C17B	1.2008 (15)	0.0178 (6)	0.2393 (6)	0.120 (2)	0.421 (7)
H17C	1.0994	0.0312	0.2241	0.144*	0.421 (7)
H17D	1.2333	0.0246	0.2849	0.144*	0.421 (7)
N1	1.0722 (2)	−0.01207 (10)	0.10293 (11)	0.0576 (6)	
N2	0.9665 (3)	−0.05905 (9)	0.10217 (11)	0.0593 (6)	
N3	0.6711 (2)	0.17352 (9)	0.02206 (10)	0.0510 (5)	
H3N	0.7140	0.2027	0.0059	0.061*	
O1	0.4373 (2)	0.24036 (8)	0.03876 (10)	0.0743 (6)	
O2	0.44423 (19)	0.14312 (8)	0.09892 (10)	0.0613 (5)	
S1	0.52632 (7)	0.19552 (3)	0.07563 (3)	0.0508 (2)	
Cl1	0.65782 (9)	−0.08137 (3)	0.07145 (5)	0.0776 (3)	

### Atomic displacement parameters (Å^2^)

**Table:**

	*U*^11^	*U*^22^	*U*^33^	*U*^12^	*U*^13^	*U*^23^
C1	0.0444 (14)	0.0511 (15)	0.0591 (16)	0.0037 (11)	−0.0066 (12)	−0.0015 (11)
C2	0.0360 (12)	0.0497 (14)	0.0424 (12)	0.0002 (10)	−0.0015 (10)	−0.0025 (10)
C3	0.0361 (13)	0.0595 (15)	0.0486 (14)	0.0021 (11)	−0.0009 (10)	−0.0027 (11)
C4	0.0324 (13)	0.0728 (18)	0.0659 (17)	−0.0071 (12)	−0.0022 (12)	−0.0017 (13)
C5	0.0449 (14)	0.0543 (15)	0.0605 (15)	−0.0097 (12)	0.0030 (12)	0.0020 (11)
C6	0.0380 (13)	0.0514 (14)	0.0437 (13)	−0.0018 (11)	0.0008 (10)	0.0035 (10)
C7	0.0326 (12)	0.0537 (15)	0.0471 (13)	−0.0013 (10)	−0.0013 (10)	−0.0012 (10)
C8	0.0372 (13)	0.0534 (16)	0.0603 (16)	−0.0031 (11)	0.0080 (11)	0.0041 (11)
C9	0.0652 (18)	0.0606 (17)	0.0625 (17)	−0.0024 (14)	0.0008 (14)	0.0087 (13)
C10	0.0681 (19)	0.099 (2)	0.0556 (17)	−0.0019 (18)	0.0005 (15)	0.0061 (16)
C11	0.0620 (18)	0.102 (3)	0.0632 (19)	−0.0170 (18)	0.0130 (16)	−0.0167 (17)
C12	0.094 (2)	0.064 (2)	0.090 (2)	−0.0176 (18)	0.006 (2)	−0.0170 (17)
C13	0.0691 (18)	0.0497 (16)	0.082 (2)	−0.0021 (14)	0.0029 (16)	0.0027 (13)
C14	0.113 (3)	0.162 (4)	0.078 (2)	−0.028 (3)	0.007 (2)	−0.041 (2)
C15	0.0471 (15)	0.094 (2)	0.086 (2)	0.0195 (15)	−0.0203 (15)	−0.0028 (16)
C16A	0.051 (5)	0.108 (3)	0.112 (3)	0.026 (6)	−0.053 (3)	−0.003 (3)
C17A	0.125 (7)	0.150 (6)	0.085 (4)	−0.008 (6)	−0.019 (5)	−0.002 (4)
C16B	0.051 (5)	0.108 (3)	0.112 (3)	0.026 (6)	−0.053 (3)	−0.003 (3)
C17B	0.125 (7)	0.150 (6)	0.085 (4)	−0.008 (6)	−0.019 (5)	−0.002 (4)
N1	0.0400 (11)	0.0674 (15)	0.0655 (14)	0.0090 (11)	−0.0081 (10)	−0.0028 (11)
N2	0.0556 (13)	0.0575 (13)	0.0649 (14)	0.0090 (12)	−0.0079 (11)	−0.0014 (10)
N3	0.0487 (12)	0.0504 (12)	0.0540 (12)	−0.0004 (9)	0.0008 (10)	0.0125 (9)
O1	0.0602 (12)	0.0682 (12)	0.0945 (14)	0.0180 (10)	−0.0207 (11)	0.0137 (10)
O2	0.0408 (9)	0.0583 (11)	0.0848 (12)	−0.0107 (8)	0.0069 (9)	0.0003 (9)
S1	0.0372 (3)	0.0478 (4)	0.0673 (4)	0.0015 (3)	−0.0050 (3)	0.0078 (3)
Cl1	0.0639 (5)	0.0553 (4)	0.1137 (7)	−0.0094 (3)	−0.0186 (4)	0.0066 (4)

### Geometric parameters (Å, º)

**Table:**

C1—N2	1.318 (3)	C12—H12	0.9300
C1—C2	1.410 (3)	C13—H13	0.9300
C1—Cl1	1.721 (3)	C14—H14A	0.9600
C2—C7	1.398 (3)	C14—H14B	0.9600
C2—C3	1.407 (3)	C14—H14C	0.9600
C3—N1	1.371 (3)	C15—N1	1.448 (3)
C3—C4	1.400 (3)	C15—C16B	1.461 (2)
C4—C5	1.359 (3)	C15—C16A	1.464 (2)
C4—H4	0.9300	C15—H15A	0.9700
C5—C6	1.407 (3)	C15—H15B	0.9700
C5—H5	0.9300	C16A—C17A	1.286 (2)
C6—C7	1.378 (3)	C16A—H16A	0.9300
C6—N3	1.441 (3)	C17A—H17A	0.9300
C7—H7	0.9300	C17A—H17B	0.9300
C8—C13	1.386 (3)	C16B—C17B	1.286 (2)
C8—C9	1.387 (3)	C16B—H16B	0.9300
C8—S1	1.763 (3)	C17B—H17C	0.9300
C9—C10	1.381 (4)	C17B—H17D	0.9300
C9—H9	0.9300	N1—N2	1.366 (3)
C10—C11	1.384 (4)	N3—S1	1.647 (2)
C10—H10	0.9300	N3—H3N	0.8060
C11—C12	1.368 (5)	O1—S1	1.4329 (18)
C11—C14	1.519 (4)	O2—S1	1.4291 (17)
C12—C13	1.380 (4)		
			
N2—C1—C2	113.4 (2)	C11—C14—H14B	109.5
N2—C1—Cl1	120.0 (2)	H14A—C14—H14B	109.5
C2—C1—Cl1	126.52 (19)	C11—C14—H14C	109.5
C7—C2—C3	120.4 (2)	H14A—C14—H14C	109.5
C7—C2—C1	136.1 (2)	H14B—C14—H14C	109.5
C3—C2—C1	103.5 (2)	N1—C15—C16B	125.2 (5)
N1—C3—C4	132.1 (2)	N1—C15—C16A	106.5 (3)
N1—C3—C2	106.4 (2)	C16B—C15—C16A	18.8 (5)
C4—C3—C2	121.5 (2)	N1—C15—H15A	110.4
C5—C4—C3	116.9 (2)	C16B—C15—H15A	98.8
C5—C4—H4	121.5	C16A—C15—H15A	110.4
C3—C4—H4	121.5	N1—C15—H15B	110.4
C4—C5—C6	122.5 (2)	C16B—C15—H15B	102.0
C4—C5—H5	118.8	C16A—C15—H15B	110.4
C6—C5—H5	118.8	H15A—C15—H15B	108.6
C7—C6—C5	121.0 (2)	C17A—C16A—C15	128.9 (8)
C7—C6—N3	119.3 (2)	C17A—C16A—H16A	115.5
C5—C6—N3	119.7 (2)	C15—C16A—H16A	115.5
C6—C7—C2	117.6 (2)	C16A—C17A—H17A	120.0
C6—C7—H7	121.2	C16A—C17A—H17B	120.0
C2—C7—H7	121.2	H17A—C17A—H17B	120.0
C13—C8—C9	120.2 (3)	C17B—C16B—C15	117.8 (10)
C13—C8—S1	120.3 (2)	C17B—C16B—H16B	121.1
C9—C8—S1	119.5 (2)	C15—C16B—H16B	121.1
C10—C9—C8	119.6 (3)	C16B—C17B—H17C	120.0
C10—C9—H9	120.2	C16B—C17B—H17D	120.0
C8—C9—H9	120.2	H17C—C17B—H17D	120.0
C9—C10—C11	121.1 (3)	N2—N1—C3	111.97 (18)
C9—C10—H10	119.5	N2—N1—C15	120.6 (2)
C11—C10—H10	119.5	C3—N1—C15	127.2 (2)
C12—C11—C10	117.9 (3)	C1—N2—N1	104.6 (2)
C12—C11—C14	122.2 (3)	C6—N3—S1	118.87 (15)
C10—C11—C14	119.9 (3)	C6—N3—H3N	115.8
C11—C12—C13	123.0 (3)	S1—N3—H3N	108.1
C11—C12—H12	118.5	O2—S1—O1	119.90 (11)
C13—C12—H12	118.5	O2—S1—N3	106.65 (10)
C12—C13—C8	118.2 (3)	O1—S1—N3	105.41 (11)
C12—C13—H13	120.9	O2—S1—C8	107.81 (11)
C8—C13—H13	120.9	O1—S1—C8	108.85 (12)
C11—C14—H14A	109.5	N3—S1—C8	107.64 (10)

### Hydrogen-bond geometry (Å, º)

**Table:**

*D*—H···*A*	*D*—H	H···*A*	*D*···*A*	*D*—H···*A*
N3—H3*N*···O1^i^	0.81	2.39	3.140 (3)	155
C4—H4···O2^ii^	0.93	2.44	3.364 (3)	171
C5—H5···O1^i^	0.93	2.58	3.282 (3)	132

Symmetry codes: (i) *x*+1/2, −*y*+1/2, −*z*; (ii) *x*+1, *y*, *z*.
