# Supplementary figures and images for: Crystal structure of N-(1-allyl-3-chloro-1H-indazol-5-yl)-4-methyl­benzene­sulfonamide
Source: Acta Crystallogr Sect E Struct Rep Online. 2014 Aug 23;70(Pt 9):o1041–2. doi: 10.1107/S1600536814018194 (PMC4186064; doi:10.1107/S1600536814018194)

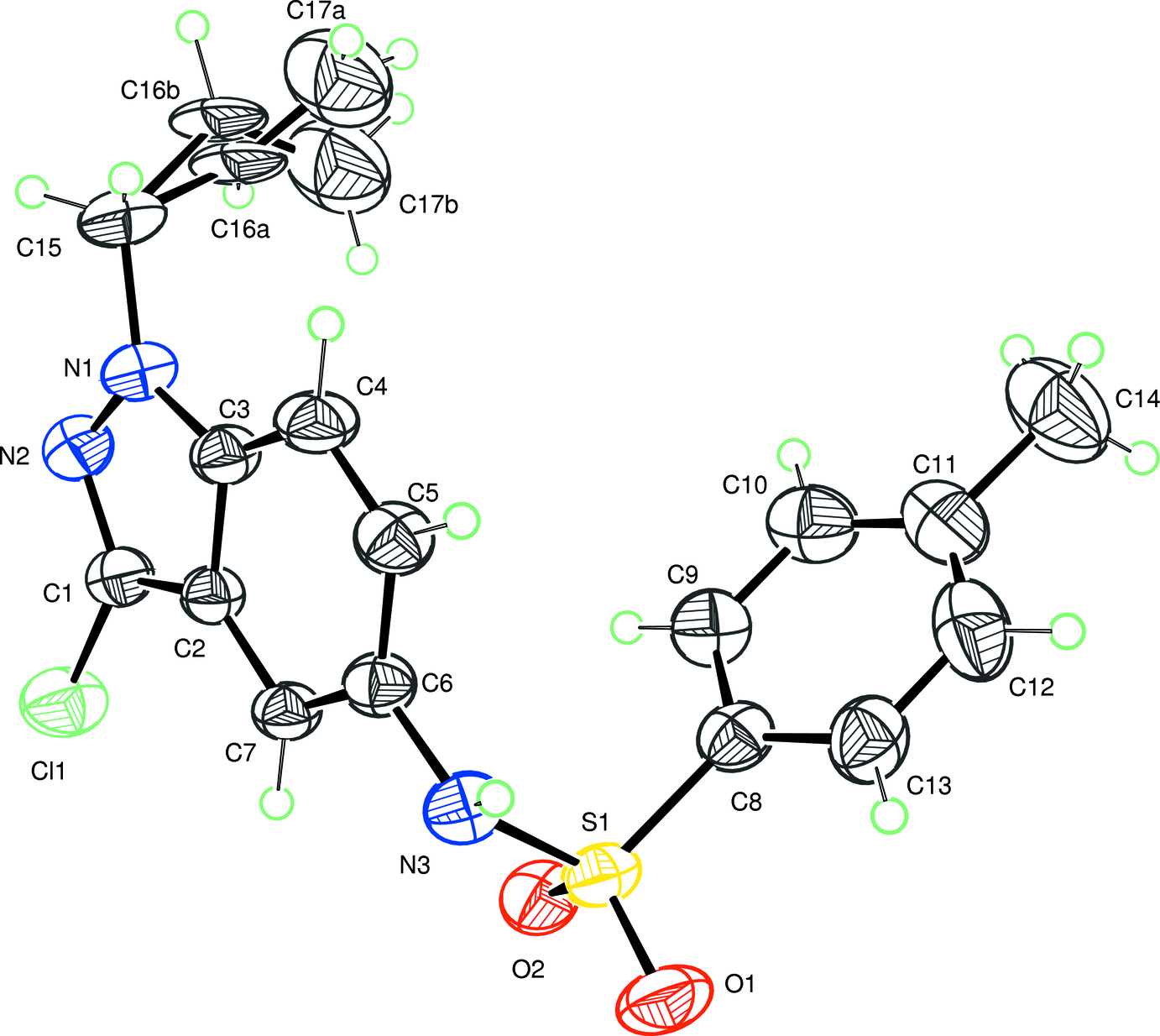

Supplement: Supplementary file 4 [file e-70-o1041-fig1.tif]

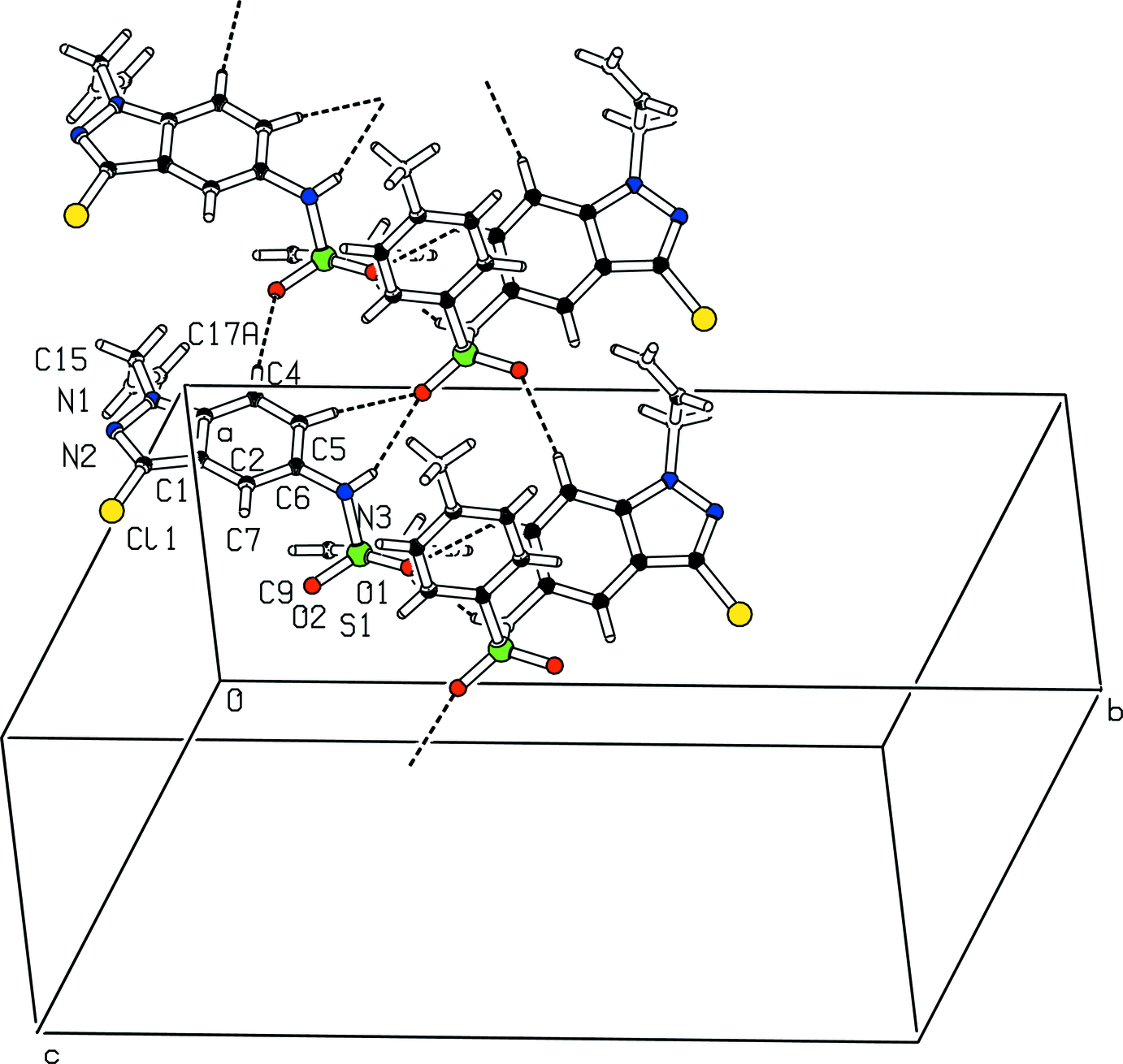

Supplement: Supplementary file 5 [file e-70-o1041-fig2.tif]
